# Flavonoids and Colorectal Cancer Prevention

**DOI:** 10.3390/antiox7120187

**Published:** 2018-12-10

**Authors:** Yanyan Li, Tao Zhang, Grace Y. Chen

**Affiliations:** 1 College of Science and Humanities, Husson University, Bangor, ME 04401, USA; liy@husson.edu; 2 Department of Basic Pharmaceutical Sciences, School of Pharmacy, Husson University, Bangor, ME 04401, USA; zhangt@husson.edu; 3 Division of Hematology & Oncology, Department of Internal Medicine, Rogel Cancer Center, University of Michigan, Ann Abor, MI 48109, USA

**Keywords:** flavonoids, chemoprevention, colorectal cancer, microbiota, inflammation

## Abstract

Colorectal cancer (CRC) is the third most common cancer, but despite advances in treatment, it remains the second most common cause of cancer-related mortality. Prevention may, therefore, be a key strategy in reducing colorectal cancer deaths. Given reports of an inverse association between fruit and vegetable consumption with colorectal cancer risk, there has been significant interest in understanding the metabolism and bioactivity of flavonoids, which are highly abundant in fruits and vegetables and account for their pigmentation. In this review, we discuss host and microbiota-mediated metabolism of flavonoids and the potential mechanisms by which flavonoids can exert protective effects against colon tumorigenesis, including regulation of signaling pathways involved in apoptosis, cellular proliferation, and inflammation and modulation of the gut microbiome.

## 1. Introduction

Colorectal cancer is the third most common cancer worldwide [1]. Despite advances made in the treatment of colorectal cancer with improvement in survival rates with chemotherapy, colorectal cancer remains the second most common cause of cancer-related mortality and metastatic colorectal cancer remains an incurable disease [1]. An effective strategy for reducing colorectal cancer-related mortality is prevention, which includes screening, and indeed, increased screening with colonoscopies has been associated with reduced incidence of colon cancer [2]. Therefore, screening for colorectal cancer is recommended for people aged 50 and over [3]. However, while the rates of colon cancer in adults aged 50 and older have declined, there has been a disturbing increase in colorectal cancer incidence in adults younger than 50, who have not been typically not screened [4]. In fact, since 1994, there has been a 51% increase in colorectal cancer in patients aged 20–49, and this increase is not clearly linked to a genetic predisposition, suggesting that other factors, such as diet and/or physical activity may underlie the increase incidence in this population [5]. For these reasons, the American Cancer Society has lowered the recommended age to start colorectal cancer screening to 45. Regardless, there is a clear need to define strategies to reduce colorectal cancer risk in addition to cancer screening.

As in most cancers, colorectal cancer is largely driven by the accumulation of genetic mutations in oncogenes and tumor suppressor genes that occur in a stepwise fashion [6,7], and therefore, increasing age and inherited mutations, such as in the adenomatous polyposis coli (Apc) tumor suppressor gene, are significant risk factors for the development of colorectal cancer [8]. This sequential accumulation of mutations is generally associated with the stepwise progression from normal intestinal epithelium to the development of a premalignant tumor, or adenoma, to a frank adenocarcinoma [6]. These mutations promote neoplastic transformation by disrupting cell biological processes that underlie the hallmarks of cancer, including evasion of apoptosis, increased cellular proliferation, and tumor invasion/metastases [9,10]. Another key promoter of colon carcinogenesis is chronic inflammation as it can lead to the production of growth factors inducing cellular proliferation, DNA-damaging oxygen radical species, extracellular matrix-modifying enzymes that can facilitate tissue invasion, and survival factors that lead to decreased apoptosis, resulting in a microenvironment conducive to tumorigenesis [10]. Indeed, patients with inflammatory bowel disease have a substantially higher risk of developing colorectal cancer than normal, healthy individuals [11]. Environmental factors can also contribute to colon carcinogenesis; specifically, physical activity, diet, and smoking, and alcohol intake can all influence colorectal cancer risk [12]. However, the mechanisms by which these activities affect colorectal cancer pathogenesis remains to be fully elucidated.

There is increasing evidence that the gut microbiome can also contribute to colorectal cancer risk, and changes in the gut microbiome are strongly linked to the diet, which in turn, has been associated with colorectal cancer risk [13,14]. The gut microbiota performs several vital functions for the human host, including dietary metabolism, colonization resistance against invasive pathogens, detoxification of potential carcinogens, and promotion of immune homeostasis [15,16]. More specifically, the gut microbiota can regulate regulatory T cell development and Th17 responses, both of which have been implicated in colon cancer progression and in development of inflammatory bowel disease, a major risk factor for colon cancer [17,18]. In addition, the gut microbiota can generate a significant number of metabolites that can have both local and systemic effects, notably secondary bile acids, which can promote colon cancer, and short-chain fatty acids (SCFAs), which have anti-inflammatory and epithelial regulatory activities that can protect against colorectal cancer development and can regulate epithelial repair and proliferation [19]. Thus, perturbations in the microbiome can affect host inflammatory and cell proliferative responses, which, in turn, can also impact colon tumorigenesis. Consistently, patients with colorectal cancer have an altered composition of the gut microbiome compared to healthy individuals, with reduced overall diversity and accumulations in certain bacterial populations including *Fusobacterium nucleatum*, entertoxigenic *Bacteroides fragilis*, and polyketide synthase-positive (*pks+*) *Escherichia coli* [20,21,22,23,24,25,26], and studies in mice have suggested that changes in the gut microbiome can directly contribute to colon tumorigenesis [27]. Thus, it is now presumed that pathologic alterations in the composition of the gut microbiome, also commonly referred to as dysbiosis, may lead to the accumulation of pro-inflammatory, pro-tumorigenic bacteria and/or the depletion of protective bacteria that have anti-inflammatory or tumor-suppressive activities [27]. How dysbiosis occurs remains unclear; however, both host genetics, inflammation, and carcinogenic stimuli, which can affect colon cancer susceptibility, can also cause changes in the gut microbiome [27,28]. Moreover, environmental and lifestyle factors that affect colorectal cancer risk, such as diet and smoking, can also potentially affect the microbiota composition [29,30]. Thus, nutritional intervention may be one strategy to modify the gut microbiome, inflammatory responses, and colorectal cancer risk.

Dietary interventions have generated significant interest as associations have been observed between consumption of certain foods and colorectal cancer risk with the strongest evidence suggesting a link between red meat consumption and increased risk, while foods containing wholegrains or dietary fiber are associated with decreased risk [12]. Diets high in fruits and vegetables have been inversely associated with many different types of cancers including colorectal cancer [31,32,33]. A class of compounds particularly abundant in these types of foods are flavonoids, and are rich in fruits, vegetables, red wine, and green tea, which have generally been associated with protective effects against cancer. In fact, a meta-analysis revealed that high intake of certain dietary flavonoids is associated with decreased risk of colon and rectal cancer [34]. However, the mechanisms by which flavonoids may suppress colon carcinogenesis remain to be fully elucidated, but have been related to their anti-inflammatory, antioxidant, and pro-apoptotic properties. In addition, certain members of the gut microbiota significantly contribute to the metabolism of flavonoids, and there is increasing evidence that flavonoids can also alter the composition of the gut microbiome, potentially giving the microbiota a central role in the bioactivity of flavonoids. In this review, the metabolism and bioavailability of flavonoids as well as potential mechanisms of action behind the protective effects against colon tumorigenesis using some of the more well-studied flavonoids as examples will be discussed.

## 2. General Overview of Flavonoids

Dietary polyphenols are natural compounds that have been used for years as nutraceuticals due to their various beneficial effects on human health. They are prevalent in fruits, vegetables, whole grains, and plant-derived beverages. Polyphenols are characterized by hydroxylated phenyl moieties with different number of phenolic rings and substituting groups. It is a large heterogeneous group of compounds which can be generally classified into flavonoids and non-flavonoids [35]. Flavonoids are the largest class of polyphenols and the most important in plant pigmentation. Aside from being pigments, flavonoids provide various biochemical functions in seed maturation, protection from different biotic and abiotic stresses, and heat acclimation and freezing tolerance, and act as detoxifying and defensive agents [36].

**Figure 1:**
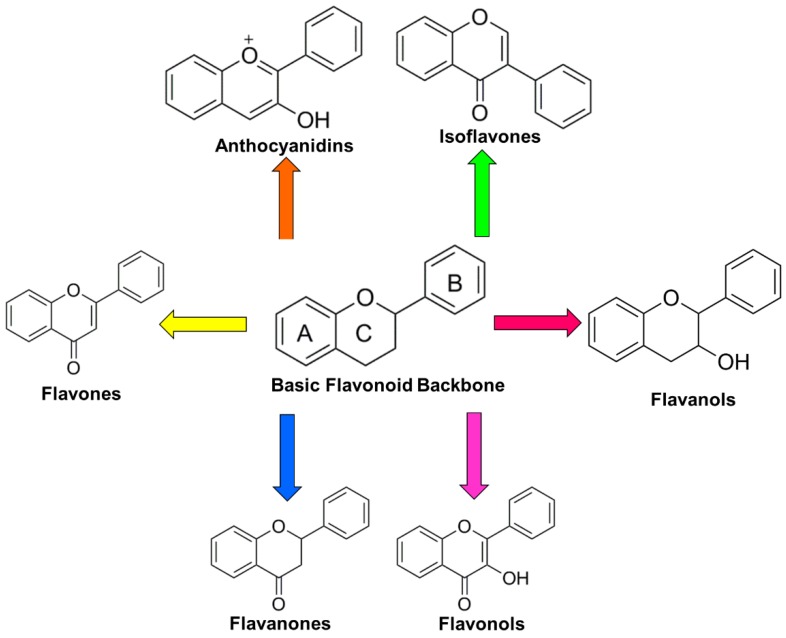
Basic structure of flavonoids and their subclasses. The core structure of flavonoids is a diphenylpropane skeleton (C6-C3-C6), which contains two phenyl rings and one heterocyclic ring. Based on their chemical structure, flavonoids can be further classified further into flavones, flavanones, flavanols, flavonols, isoflavones, and anthocyanins/anthocyanidins.

There are over 9000 flavonoids that have been described [37]. The core structure of flavonoids is a diphenylpropane skeleton (C6-C3-C6), which contains two phenyl rings and one heterocyclic ring (Figure 1) [38]. Their antioxidant capacity has largely been attributed to the presence of multiple 3- and 5-hydroxyl groups as well as 3′- and 4′-catechol groups [39]. Based on their chemical structure, flavonoids can be classified further into six major subclasses: flavones, flavanones, flavanols, flavonols, isoflavones, and anthocyanins/anthocyanidins (Figure 1) [40,41]. Other flavonoids may include compounds such as biflavonoids (e.g., ginkgetin), flavanonols (e.g., taxifolin), prenylflavonoids (e.g., artocarpin), flavonolignans (e.g., silibinin), glycosidic ester flavonoids, and chalcones [40,42]. In plants, the majority of flavonoids exist in the form of glycosides [43].

### 2.1. Flavones

Flavones are a group of flavonoids characterized by a double bond between C-2 and C-3 in the flavonoid skeleton, and a non-saturated C-3 chain. Flavones are widely distributed among the higher plants and play a variety of important roles. They are the primary pigments or co-pigments in white and blue flowers, respectively [44]. The major flavones studied are apigenin and luteolin. Apigenin is one of the most prominent flavone aglycones. It is commonly found in vegetables, herbs and plant-derived beverages such as wine, beer, and chamomile [45,46]. Celery, artichokes, and parsley also contain high amounts of apigenin [47], while apigenin-7-glucoside (A7G) is present at high levels in red wine, artichokes and chamoumile [47]. Apigenin has shown prominent antibacterial, anti-inflammatory and antispasmodic effects [43]. Luteolin, like apigenin, occurs in many vegetables and fruits such as broccoli, celery, carrots, parsley, cabbages, peppers and apple skins [43]. As an antioxidant that scavenges reactive oxygen species (ROS) and as a pro-oxidant due to auto-oxidation, luteolin exhibits anti-cancer, anti-allergy, and anti-inflammatory effects [43].

### 2.2. Flavanones

The backbone structure of flavanone is 2,3-dihydro-2-phenylchromen-4-one [41]. Flavanones represent one of the largest subgroups of flavonoids. They are extensively disseminated in plants and especially rich in *Citrus* species. In the diet, orange juice is the foremost food that provides flavanones. Common flavanones are the aglycones such as naringenin, hesperetin, eriodictyol, isosakuranetin, and their respective glycosides [41]. Naringenin and its derivatives are typically found in grapefruit and sour oranges, while hesperetin and its derivatives are characteristic flavanones of sweet oranges, tangelos, lemons and lime [40]. Flavanones, of which naringenin has been studied the most extensively, are biologically active with antioxidant, anti-inflammatory, and anti-microbial activities [43,48,49]. The antioxidant activity of flavanones is highly dependent on a hydrophilic environment and the presence of a catechol group [43].

### 2.3. Flavanols

Flavanols constitute a very complex group of flavonoids with the backbone structure of 2-phenyl-3,4-dihydro-2H-chromen-3-ol, also known as flavan-3-ol [41]. Flavanols exist primarily in tea, wine, cocoa, apples and fruit juices or jams [41]. Catechins, which are known as the major building blocks of tannins, are the most important representatives of flavanols. Catechins are found abundantly in green and black tea. Different types of catechins include catechin, gallocatechin, catechin 3-gallate, gallocatechin 3-gallate, epicatechin, epigallocatechin, epicatechin 3-gallate, and epigallocatechin 3-gallate (EGCG) [43]. Proanthocyanidins are dimers, oligomers, and polymers of catechins, which are known as a type of condensed tannin [50].

### 2.4. Flavonols

Flavonols are a class of flavonoids with a 3-hydroxy-2-phenylchromen-4-one backbone [41]. This class of flavonoids are well-known for their antioxidant properties and other biological activities [43]. They are the most common flavonoids in fruit and vegetables, accumulating mainly in skin and leaves. Onions, leeks, kale, apples, berries, grapes, and grape products are all major food sources of flavonols [43]. The main representatives of flavonols are quercetin, kaempferol, myricetin, and isorhamnetin.

Quercetin is found in onion, broccoli, apple, tea and red wine [46]. It is a water-soluble plant pigment with high antioxidant and anti-inflammatory activity [51,52,53,54]. Kaempferol is a flavonol antioxidant occurring in spinach, kale and broccoli [43]. It has been shown to regulate various signaling protein related to angiogenesis, apoptosis, metastasis and inflammation [55]. Myricetin is commonly found in berries, vegetables and in plant-derived teas and wines [56]. It occurs naturally in both free and glycosidically-bound forms and is poorly soluble in water [57]. Myricetin exhibits a wide range of biological activities including antioxidant, anti-cancer, anti-diabetic, and anti-inflammatory effects [56]. Isorhamnetin is the 3-methyl metabolite of quercetin but also occurs naturally in plants. It is extracted from herbal medicines such as *Persicaria thunbergii* H. and *Hippophae rhamnoides* L. [58]. Although it is less well-studied compared to quercetin, it has been demonstrated to have anti-cancer, anti-viral, and antioxidant effects [58,59,60,61].

### 2.5. Isoflavones

Isoflavones possess a 3-phenylchromen skeleton which is derived from the 2-phenylchromen system [43]. Soybeans and soy foods are the richest source of isoflavones [41], and red clover and kudzu also contain high amount of isoflavones [62]. Genistein and daidzein are the two major isoflavones, and have been studied extensively. Genistein, known as a phytoestrogen, can modulate steroid hormone receptors and multiple metabolic pathways, making it an important dietary ingredient that can prevent and treat common disorders [63]. Daidzein, another isoflavone present in soy, is an inactive analog of genistein. Daidzein is also a phytoestrogen that binds to estrogen receptors with both weak estrogenic and weak anti-estrogenic effects [64].

### 2.6. Anthocyanins and Anthocyanidins

Anthocyanins and anthocyanidins are a group of water-soluble pigments with significant antioxidant activity responsible for the blue, red, purple, and orange colors present in many fruits and vegetables, such as red-skinned grapes, apples, pears, radishes, and red/purple cabbage [65,66,67]. Anthocyanidins have the backbone structure of 2-phenylchromenylium [41], and are formed by the addition of glycose (mainly glucose), acyl, hydroxycinnamic acid or other moieties to the main structure of major anthocyanidins [68]. Anthocyanin, for example, is the glycoside form of the anthocyanidin, aglycone. Cyanidin, pelargonidin, delphinidin, malvidin, petunidin, and peonidin are the most commonly found anthocyanidins [65]. Cyanidin is a strong antioxidant present in most red colored berries such as bilberry, blackberry, blueberry, cherry, cranberry, elderberry, hawthorn, loganberry, and raspberry, and in other fruits such as apples, pears, peaches, and plums [65,69]. Pelargonidin is an anthocyanidin that produces an orange color [65] and is present in all berries, but primarily strawberries [70]. Delphinidin is an anthocyanidin that provides the blue-red colors of flowers, fruits, and red wine [65] while malvidin is responsible for the pigments in red grapes and blueberries. Malvidin possesses significant antioxidant capacity and exhibits anti-inflammatory effects [66,67]. Petunidin is an O-methylated anthocyanidin derived from delphinidin and provides blue-red pigments to flowers, fruits, and red wine. Peonidin is also an O-methylated anthocyanidin that gives purplish-red hues to flowers such as the peony as well as berries and vegetables.

## 3. Host Metabolism of Flavonoids

The bioavailability of flavonoids is generally poor, but can vary significantly among different classes as well as individual flavonoids [71]. Factors that affect bioavailability and absorption of a flavonoid include its molecular weight, the nature of glycosylation and metabolic conversion by host conjugating enzymes, and the composition of the gut microbiota [71]. Flavonoids occur in plants in several different forms, such as aglycones, esters, and glycosides [72] although the majority of plant flavonoids exist as glycosides [73,74,75]. The sugar moiety is coupled to the aglycone as O-glycosides via a hydroxy group and less frequently as C-glycosides [75]. After ingestion, flavonoid glycosides are absorbed in the small intestine where they are deglycosylated and further conjugated, while the rest pass into the colon where they are metabolized by the gut microbiota [73,76,77,78,79]. Two enzymes in the human small intestine have been identified to deglycosylate flavonoids [80,81,82]. Lactase-phlorizin hydrolase, a brush border enzyme, was reported to hydrolyze O-glucosides of quercetin, genistein, and daidzein in vitro [80]. Cytosolic beta-glucosidase in the enterocytes was reported to hydrolyze O-glucosides of quercetin, genistein, daidzein in cell-free extracts from human intestine [83].

Once deglycosylated, the produced flavonoid aglycons enter the intestinal epithelial cells, where phase II enzymes catalyze conjugation reactions [82]. Three types of phase II enzymes—uridine-5’-diphosphate-glucuronosyltransferases, sulfotransferases, and catechol-o-methyltransferases—have been identified that are capable of metabolizing flavonoids [82,84,85,86]. The conjugated flavonoids are subsequently absorbed into the circulation and transported to the liver where they undergo additional conjugation such as methylation and sulfation [82,87]. These flavonoid metabolites can then circulate systemically and exert their biological effects, or can return to the intestine via the bile [75,82,86,87,88,89,90]. Upon return into the intestine, flavonoid metabolites can be deconjugated by the gut microbiota and reabsorbed or act locally in the tissue [75,82,91]. This recycling of flavonoids through the enterohepatic circulation contributes to the improved plasma levels of flavonoids in humans [92].

## 4. Microbial Metabolism of Flavonoids

As many flavonoid glycosides are poorly absorbed in the small intestine, resulting in substantial quantities in the colon [73,76,77,78], the gut microbiota can have a crucial role in the biotransformation of flavonoid glycosides. The diversity of the gut bacteria and enzymes they contain allow the formation of a variety of bioactive metabolites from flavonoids with varying anti-inflammatory, antioxidant, and anti-tumor activities [82,93,94]. For example, microbial metabolites of anthocyanins have been shown to affect the proliferation and viability of colon cancer cell lines in vitro although the concentrations used were quite high in the 10–100 µM range [95,96]. Thus, a better understanding of the bacterial populations and activities in flavonoid metabolism and bioavailability will be important to harness the beneficial effects of flavonoids for colorectal cancer prevention.

Flavonoids can be extensively metabolized into a range of products by the gut microbiota. Intestinal bacteria, including specific species such as *Clostridium*, *Eubacterium*, *Lactococcus*, and *Parabacteroides*, have the capacity to catalyze O-deglycosylation, C-deglycosylation, demethylation, dehydroxylation, ester cleavage, reduction of carbon–carbon double bonds, isomerization, ring fission, extension and truncation of the aliphatic carbon chain, and decarboxylation [75]. Heterogeneity in the composition of the gut microbiome may result in very different profiles of flavonoid metabolites between individuals as exemplified by a study of the metabolite profiles obtained from in vitro incubations of fecal-derived human microbiota from 10 different human subjects with extracts from black tea and a mixture of red wine and grape juice. There were significant inter-individual differences in the types and levels of metabolites produced [75,97], likely reflecting differences in bacterial populations and their respective activities between individuals.

Deglycosylation, the hydrolysis of glycosidic bonds, is usually the first step of microbial conversion of flavonoids to produce flavonoid aglycons. While some human glycosidases may hydrolyze O-glucosides, deglycosylation of C-glucosides and other C- and O-glycosides can only by performed by gut bacteria [75]. Specific human gut bacteria have been reported for O-deglycosylation of flavonoid glycosides, including a number of *Bifidobacterium* species [50,98,99], *Lactobacillus* species [50,98,99], *Enterococcus* species, and *Bacteroides* species [100,101,102,103]. The deglycosylation of flavonoid C-glycosides has not been well-investigated thus far. Some members of *Lachnospiraceae*, *Enterococcaceae*, and *Streptococcaceae* were reported to cleave the C-coupled flavone and isoflavone glucosides [103,104,105,106,107].

The flavonoid aglycons can be further metabolized into a variety of metabolites [82]. The phenolic rings of flavonoids often carry hydroxy- and methoxy- groups [75], and either dehydroxylation or demethylation can occur via the enzymatic activity of *Eubacterium, Blautia, Eggerthella, Adlercreutzia, and Escherichia* [108,109,110,111,112,113,114]. Moreover, some human gut bacteria, for example, *Eubacterium ramulus* and *Flavonifractor plautii*, can also degrade aglycones of the different flavonoid subtypes into small molecules with bioactivity [75,79,100,115,116,117,118,119,120,121]. These metabolites include various ring-fission products. For example, the B-ring of the flavanonols may be converted into hydroxyphenylacetic acids and the A-ring into short-chain fatty acids (SCFAs) [75]. The C-ring of the flavanones may be transformed into dihydrochalcones [75]. Degradation of isoflavone aglycones may result in equol or O-desmethylangolensins, which may be further cleaved into small phenolic products [75,82]. These metabolites are generally bioavailable from the colon, and may have potent activities locally and systematically. Whether the relative abundances of specific bacterial populations in the gut determine flavonoid metabolite profiles and the relative potency of these metabolites in mediating colorectal cancer risk remains to be determined. Intriguingly, quercetin, for example, can be metabolized and degraded into the SCFAs acetate and butyrate, which have been associated with cytoprotective effects in the intestinal epithelium and protection against colon tumorigenesis [79,122,123]. Thus, it is possible that the efficacy of a flavonoid is largely dependent on the ability of an individual’s microbiota to generate specific bioactive metabolites with anti-tumor activity.

## 5. Anti-Colorectal Cancer Effects of Major Flavonoids

### 5.1. Anthocyanidins

Anthocyanins and their metabolites, anthocyanidins, provide a variety of health benefits attributed to their antioxidant, anti-inflammation, and anti-cancer activities [39,41,124,125,126]. Anthocyanins have been reported to reduce both colorectal cancer and inflammatory bowel disease, a major risk factor for the development of colorectal cancer. Its protective activities have been largely attributed to its ability to negatively regulate inflammatory signaling pathways including nuclear factor kappa light chain enhancer of activated B cells (NF-κB), mitogen-activated protein kinase (MAPK), c-Jun N-terminal kinase (JNK) and signal transducer and activator of transcription (STAT). It is also capable of inhibiting cell proliferative pathways such as the Wnt signaling pathway, which is upregulated in the majority of sporadic colorectal cancers [79,127]. The anthocyanidins delphinidin and cyanidin, for example, have been shown to have direct cytotoxicity against metastatic colon cancer cell lines in vitro [128], leading to apoptosis. This was not necessarily related to its antioxidant effects as both, in fact, acted as pro-oxidants with ROS accumulation in tumor cells that may increase oxidative stress and induce an apoptotic response [129]. Interestingly, this effect was not observed for all anthoycanidins, as malvidin (the 3′,5′-methoxy derivative of delhinidin) and pelargonidin did not have any anti-tumor activity and moreover, the protective effect of delphinidin and cyanidin was not observed with all colorectal cancer cell lines. Delphinidin, in particular, upregulated the expression of p53, which can induce cell cycle arrest and apoptosis [130]. Consistently, there was increased expression of the pro-apoptotic factor B-cell lymphoma 2-associated X protein (Bax) and concomitant decrease in expression of the anti-apoptotic factor (B-cell lymphoma 2) Bcl-2 associated with inhibition of NFκB, a regulator of Bcl-2 and Bax expression [130]. Bcl-2, the anti-inflammatory effect of anthocyanin was confirmed in a mouse model of chemically-induced colitis by dextran sulfate sodium (DSS), a chemical which directly injures the intestinal epithelium causing a bacterial-driven inflammatory response, using either 1 or 10% anthocyanin extract derived from bilberries [131]. In addition, treatment of Balb/c mice with a 10%, but not 1%, anthocyanin-rich extract reduced the number of tumors that developed in a mouse model of inflammation-associated colon tumorigenesis in which mice are first injected with an experimental carcinogen, azoxymethane (AOM), followed by multiple rounds of water containing dextran sulfate sodium (DSS) to induce chronic inflammation that mimics inflammatory bowel disease in humans (AOM/DSS model), although there are features of this model that also recapitulate human sporadic colon cancer [131,132]. In another study using the AOM/DSS model, C57BL/6 mice fed an extract containing black raspberry anthocyanins (purity >90%), consisting mostly of cyanidin-O-glucoside, cyanidin-O-xylosylutinoside, and cyanidin-O-rutinoside, reduced both inflammation and tumor numbers compared to that of control mice. Interestingly, the addition of anthocyanin extracts reversed potentially pathologic changes in the gut microbiome as determined by terminal restriction fragment length polymorphism (T-RFLP) analysis after AOM/DSS treatment, namely a reduction in the pro-inflammatory *Enteroccoccus* species while increasing *Eubacterium rectale*, *Faecalibacterium prausnitzii*, and *Lacobacillus* that have typically been associated with anti-inflammatory and anti-proliferative capabilities [133]. In addition, changes were observed in the expression of the DNA methyltransferases DNMT31 and DNMT3B, that can lead to promoter demethylation of genes involved in tumor suppression, for example, secreted frizzled-related protein 2 (SFRP2), an antagonist of the Wnt pathway, which is disrupted in the majority of colorectal cancers [127,133]. Consistently, an effect on demethylators was also observed in another study after addition of black raspberry-derived extract to various colon cancer cell lines [134]. Although the dose of extract used was relatively high, another study using a bilberry extract resulting in a dietary anthocyanin content of 0.3%, an amount more achievable in humans, resulted in reduced adenoma formation in Apc^Min^ mice, which harbor a mutation in the *Apc* tumor suppressor gene that commonly occurs in human sporadic colon cancers [135,136].

The most compelling evidence of a protective anti-inflammatory and anti-proliferative effect for anthocyanidin came from human studies in which the ingestion of an anthocyanidin-rich bilberry extract ameliorated colitis and was associated with reduced NFκB activation and production of pro-inflammatory mediators in colon biopsies from inflammatory bowel disease patients [137,138] and reduced cellular proliferation in tumor samples from colorectal cancer patients [139]. Consumption of black raspberry powder was also associated with changes in methylation status of various tumor suppressor gene promoters, increased apoptosis, and decreased surrogate markers of cellular proliferation in biopsies of colorectal cancers and normal adjacent tissue [140]. These studies, however, were small and also involved extracts and freeze-dried black raspberry powder, and therefore can contain numerous other bioactive compounds with anti-tumor activity. Therefore, additional studies in a larger population with purified compounds will be needed to determine more precisely the anti-tumor effects of particular anthocyanidins.

### 5.2. Apigenin

Apigenin, one of the flavones, has multiple activities that promote its anti-colorectal cancer effect. Although there have been no clinical trials conducted to date evaluating the effects of apigenin supplementation on colorectal cancer prevention, in one prospective study, 87 high-risk patients who either had a history of resected colorectal cancer or a polypectomy for an adenoma were given a flavonoid mixture consisting of 10 mg apigenin and 10 mg epigallocatechin-gallate or nothing over a period of 2–5 years with 67% of treated patients taking the flavonoid supplement more than 1 year with no adverse side effects. Although there was no statistically significant difference in colon cancer or adenoma recurrence rates between the treated and control groups, there was a trend for more favorable outcomes in the treatment group with cancer recurrence in 20% of controls and none in the treated group. However, the combined recurrence for neoplasia (cancer and adenoma) was significantly lower in the treatment group compared to that of the control group [141]. In addition, in the Polyp Prevention Trial, a dietary intervention trial in which 2079 subjects were randomized to either a control arm or to a low-fat, high fruit and vegetable diet, high apigenin intake was inversely associated with advanced adenoma recurrence [142].

Most data supporting the protective effect of apigenin against the development of colorectal cancer are largely from preclinical studies using colorectal cancer cell lines and animal models. Apigenin can induce G2/M cell cycle arrest of multiple colon cancer cell lines including SW480, HCT116, HT-29, and Caco-2 to varying degrees, which was associated with decreased expression of cyclin B1 proteins and the cyclin dependent kinase p34(cdc2) [143,144,145,146]. The induction of apoptosis may be related to its ability to its pro-oxidative effect, leading to increased ROS production and oxidative stress [147]. Like anthocyanidins, apigenin can also promote apoptosis by inducing the expression of p53 and altering the Bax/Bcl-2 ratios [148,149].

Apigenin can also affect multiple signaling pathways involved in cellular proliferation. For example, apigenin can inhibit Wnt signaling in colorectal cancer cells in vitro, possibly through an autophagy-dependent pathway with subsequent downregulation of Wnt target genes such as cyclin D1 and c-myc that are involved in colon epithelial proliferation [150,151,152]. Other inflammatory and cell proliferative pathways such as MAPK and extracellular signal-regulated kinase (ERK) can be affected by apigenin in colon cancer cells although how these different pathways interact to affect colon tumorigenesis remains to be fully elucidated [153].

In vivo studies using mouse models of colon cancer have not yielded consistent robust anti-tumor effects. In CF-1 mice injected with either a single dose of azoxymethane for 4 or 6 weeks to induce aberrant crypt foci, a lesion believed to precede the development of carcinoma, only 0.025%, but not 0.1% apigenin-fed mice exhibited a reduction in tumor incidence [154]. In the ApcMin mouse model, there was also no significant difference, although the relevance of this model for human colon cancer given the predominance of tumors in the small intestine has been disputed [136,154,155]. However, in rats injected with AOM, a significant decrease in the number of aberrant crypt foci was observed in the group fed 0.1% apigenin diet group for 10 weeks prior to AOM injection, associated with reduced proliferating cells and increased apoptosis [156]. Whether the discrepancy in results from results reflect differences in the animal model used, the diet preparation, the purity of apigenin, and length of exposure remains to be determined.

It is possible, however, that maximal effects of apigenin may be more evident in the context of inflammation given its ability to inhibit pro-inflammatory pathways [157,158]. Consistently, in both models of inflammatory bowel disease and colitis-associated colon cancer, mice exhibited reduced NFκB activation and STAT3 activation, which has been linked to increased epithelial proliferation [158], and decreased expression of pro-inflammatory cytokines and chemokines [159]. Additional insight into the ability of apigenin to inhibit inflammation and inflammation-induced colon tumors was provided by another study which implicated a role for the gut microbiota and innate immune signaling. In particular, when C57BL/6 mice treated with apigenin were cohoused with a second cohort of wildtype control mice not exposed to apigenin to allow microbiome transfer between mice, the control mice were similarly protected from DSS-induced colitis as the apigenin-treated mice, suggesting that the protective, anti-inflammatory effect of apigenin was mediated in part by the gut microbiota [160]. Indeed, it was demonstrated that ingestion of apigenin was associated with changes in the composition of the gut microbiome, namely, an expansion of *Rikenellacae*, *Bacteroidales*, and *Bacteroides*, and depletion of *Clostridium* and *Lachnospiraceae*, a phenotype similarly observed in control mice after cohousing. Interestingly, these changes were not observed in apigenin-treated mice deficient in the innate immune receptor NLRP6, and consistently, apigenin-treated NLRP6-deficient mice were more susceptible to DSS-induced colitis compared to apigenin-treated wildtype mice, strongly suggesting a role for NLRP6 in mediating the protective effect of apigenin as well. In fact, apigenin was capable of inducing NLRP6 expression within the intestinal epithelium. NLRP6 is a member of the Nod-like receptor (NLR) family of innate immune receptors that are capable of sensing microbes and tissue damage and has been shown to be important for regulating the composition of the gut microbiota and for protecting against the development of colitis as well as inflammation-associated tumorigenesis [28,161,162,163,164]. The mechanism by which NLRP6 regulates microbiome composition remains unclear although it appears to be dependent on IL-18, which can upregulate expression of antimicrobial proteins [28,161]. Consistently, mice deficient in the antimicrobial peptide Reg3III that is regulated by NLRP6 are also not protected from colitis after treatment with apigenin [160]. An effect of apigenin on gut bacteria was also verified in vitro in which apigenin was added to anaerobic cultures of human fecal microbiota as well as single bacterial isolates, which affected the growth and gene expression of certain bacteria [165]. How these effects ultimately translate into protection against colon tumorigenesis remains to be determined; clearly more studies are needed to clarify the relative importance of the different apigenin-protection mechanisms in colon cancer suppression.

### 5.3. Quercetin

As with other classes of flavonoids, the flavonol quercetin has been demonstrated to have anti-tumor activity against colon cancer cells both in vitro and in vivo. Mechanisms for its cytotoxic activity against colon cancer cells include induction of apoptosis via activation of p53 and inhibition of NFκB [166,167], cell cycle arrest as a result of downregulation of cell cycle genes [168,169,170], and suppression of inflammation via downregulation of Cox2, which is commonly upregulated in colon cancer [171]. Quercetin is also capable of regulating multiple signaling pathways involved in inflammation and cellular proliferation such as the Wnt pathway [169,172], NFκB, PI3K, MAPK, and protein kinase B (Akt) [166,173]. Another potential mechanism by which quercetin can affect cellular proliferation of colon cancer cells is by upregulating expression of the G-protein coupled cannabinoid receptor, CB1-R, which in turn can bind to quercetin, resulting in inhibition of cell growth and migration via Wnt, PI3K, Akt, and STAT3 pathways [174]. This effect was abrogated in the presence of a CB1-R antagonist.

Consistent with its in vitro effects, there are multiple studies that demonstrate the efficacy of quercetin in reducing tumor numbers in both mice and rats treated with azoxymethane [175,176,177,178]. Quercetin has also been shown to stimulate the growth of specific bacteria when exposed to human fecal microbiota in vitro with increases in the relative abundance of bacteria belonging to the *Actinobacteria, Firmicutes,* and *Bacteroides* phyla [179]. In vivo, alterations in the gut microbiome associated with quercetin treatment have also been reported [180]. Specifically, in an adoptive T-cell transfer model of colitis in which T cells depleted of regulatory T cells were transferred into T-cell deficient *Rag2*^−/−^ as well as the DSS chemically-induced colitis model, administration of quercetin resulted in amelioration of colitis that was associated with macrophages expressing an anti-inflammatory gene signature [180]. In addition, quercetin treatment resulted in reduced abundance of *Proteobacteria*, which commonly blooms in colitis and during inflammation and in patients with inflammatory bowel disease (IBD) [181,182], as well as an increase in *Bacteroides* and *segmented filamentous bacteria* (*SFB*), which promotes pro-inflammatory Th17 responses [183]. Decreases in *Actinobacteria* were also noted, which is inconsistent with what was observed in vitro and may reflect greater microbial complexity in vivo [179,180]. However, these changes were observed on day 45 after adoptive transfer when colitis is already present, and therefore, it is possible that the observed alterations in the composition of the gut microbiota reflect the severity of inflammation rather than a direct effect from quercetin. Whether quercetin induces changes in the gut microbiota that are beneficial prior to the onset of inflammation remains to be determined.

To date, there are no studies that demonstrate positive effects of quercetin supplementation on colorectal cancer risk in humans. Its potential chemopreventive effects have largely been extrapolated from case-control studies correlating quercetin content based on food frequency questionnaires, that may not have accurately quantified all food sources of flavonoids, with colorectal cancer incidence. For example, in an Italian study, consumption of flavonols of which quercetin, myricetin, and kaempferol were the major constituents evaluated significantly decreased the risk of colorectal cancer [184]. In the Polyp Prevention Trial, although high intake of flavonols, including quercetin, was associated with a significant decreased risk in advanced adenoma recurrence, there was no association between quercetin alone and adenoma recurrence [142]. In addition, in the Women’s Healthy Study and Health Professionals Followup Study, two prospective cohort studies consisting of 71,976 and 35,425 evaluable women and men, respectively, there was no association between quercetin intake or even total flavonoid intake with colorectal cancer risk, although the authors of the study noted that the questionnaire used was not designed to accurately assess flavonoid intake and that dietary changes may not have been captured with a one-time questionnaire [185]. It is also possible that the dietary source of quercetin may be important as a case–control study demonstrated that increased quercetin intake was associated with a small reduction in risk of proximal colon, but not distal colon cancers, and this effect was observed only with high fruit, but low tea intake [186]. This suggests that the food source of quercetin may affect bioavailability. Indeed, it was demonstrated that higher plasma concentrations of quercetin was achieved from the consumption of onion powder as compared to apple peel powder, and similarly, higher bioavailability was achieved with ingestion of quercetin-enriched cereal bars compared to powder filled capsules [71,187,188]. Alternatively, the presence of other bioactives in foods may interact with quercetin and further modulate tumor risk. Thus, additional studies will be needed to understand the relationship between quercetin intake and colon cancer.

### 5.4. Epigallocatechin-3-Gallate

The beneficial effect of tea has largely been attributed to its epigallocatechin-3-gallate (EGCG), rather than epicatechin and epigallocatechin, content [41]. EGCG is the main flavonoid in green tea (~10–15% in an extract from green tea leaf), and there have been several studies demonstrating a protective effect of green tea against the development of recurrent adenomas and colon cancer [189,190,191,192]. Although these effects are likely not entirely due to the activity of EGCG alone, in vitro studies suggest a variety of anti-tumor mechanisms similar to those reported for other flavonoid subtypes. EGCG can inhibit not only the growth of colon cancer cells, but also their spheroid forming ability, an indicator of stem cell function [193,194]. This may, in part, be due to its ability to downregulate Wnt signaling [195], since activation of the Wnt pathway by lithium chloride can reverse the effects of EGCG and its ability to induce apoptosis [194]. EGCG can also inhibit Akt and NFκB pathways leading to reduced cyclooxygenase-2 (Cox-2) expression [196]. Moreover, although its hydroxyl groups can act as potent scavengers of reactive oxygen species, EGCG, as well as other flavonoids, can also undergo auto-oxidation to generate oxidizing radicals which, in turn, can act as a stress signal to activate pathways such as JNK to initiate cyctochrome c release and apoptosis [197,198,199]. Although it has been debated whether auto-oxidation is a phenomenon that occurs only in vitro as oxygen levels within tissues tend to be lower than that of culture media, at least one study demonstrated increased oxidative stress in lung xenograft tumors in the presence of EGCG [200,201]. Nonetheless, the relative antioxidant and pro-oxidant properties of EGCG and what actually occurs in vivo to affect tumor susceptibility will require further study. EGCG can also affect DNA methylation by regulating the expression of DNA methyltransferases [202]. For example, EGCG administration to HCT116 colon cancer cells can lead to the downregulation of DNMT3a gene expression as well as increased degradation of the protein [203]. Consistently, EGCG treatment resulted in reduced methylation of colon cancer-related genes, such as RXRα, Apc, and hMLH1 [204]. However, it remains unclear whether this is a predominant mechanism by which EGCG contributes to inhibition of colorectal carcinogenesis as the data is correlative only.

Animal studies indicate that EGCG is capable of reducing colon tumorigenesis. In rats, EGCG reduced the number of tumors that developed with AOM treatment [205,206]. The decreased tumorigenesis was associated with reduced Wnt signaling and Cox2 expression [205]. In mice, EGCG administration resulted in a significant decrease in tumors after treatment with AOM/DSS that was associated with changes in the microbiota at 8 weeks, namely *Bifidobacterium* and *Lactobacillus* (that have potential anti-inflammatory effects) were increased. As inflammation itself can alter the composition of the gut microbiota, whether the observed changes in abundance in certain bacterial populations with EGCG directly contribute to tumor suppression or merely reflect changes in the severity of inflammation remain to be determined and can be better addressed using gnotobiotic mice. In humans, the consumption of green tea resulted in changes in the gut microbiome, including increases in short-chain fatty acid-producing bacteria such as *Lachnospiraceae*, *Bifidobacteriaceae*, and *Ruminococcaceae*, and decreases in potentially proinflammatory bacteria, such as *Prevotella*, which was reported to be increased in patients with colorectal cancer [20,162,207,208]. However, whether these changes are due to EGCG and responsible for its protective effects is not known.

## 6. Conclusions

Despite the mounting evidence that high consumption of fruits and vegetables are inversely associated with colorectal cancer risk, observational studies have not provided consistent results regarding the relationship between flavonoid intake and colorectal cancer risk. For example, a recent large European cohort study consisting of 477,312 adults showed no association between intake of individual flavonoid subclasses as estimated by dietary questionnaire with colorectal cancer risk after an average of 11 years of follow-up [209] (Table 1). On the other hand, a meta-analyses of 18 cases involving 559,486 participants with 6–26 years of follow-up showed a potential colon cancer risk-reducing effect of specific flavonoid subclasses [210]. Differences in outcomes may, in part, reflect the limitations in the dietary questionnaires used and available food databases to accurately estimate flavonoid content as well as the significant inter-individual genetic and microbiome differences that exist among human subjects. However, multiple in vitro and in vivo preclinical studies, as reviewed above (Table 2), have demonstrated anti-tumor effects of specific flavonoids. The discrepancy between observational and preclinical studies may be due to the dosing of flavonoids in rodents and in cell culture that may not be achievable through the diet in human studies and more importantly, highlights the difficulty in translating preclinical data to clinical trials. Regardless, results from a limited number of small intervention trials, such as the Polyp Prevention Trial, hold promise for flavonoids as colon cancer chemoprevention agents [142].

Thus, much remains to be learned about the specific effects of the different flavonoids and their respective metabolites on colon cancer progression, which may allow the design of a nutritional intervention that optimizes the concentrations of the most bioactive compounds. Complicating this problem further is the ability of flavonoids and other dietary components to affect the growth of specific bacterial populations, which, in turn, can impact the metabolism and bioavailability of flavonoids as well as influence the development of colorectal cancer.

Future studies that combine bacterial transcriptomics and metagenomics with metabolomics will, therefore, be critical in defining specific bacterial activities that are important for the generation of specific flavonoid bioactives or are altered by the ingestion of flavonoids. Studies involving fecal transplant of microbiota associated with the intake of specific flavonoids or the administration of synthetic bacterial communities effective in flavonoid metabolism in germfree mice will provide additional evidence that flavonoid-mediated changes in the gut microbiome can be beneficial to the host and regulate colorectal cancer susceptibility. The use of germfree mice will also be important in identifying microbiome-specific contributions to the anti-tumor effects of flavonoids. Since there is significant inter-individual heterogeneity in the composition of the gut microbiota [211], interventions to modulate the gut microbiome to enhance the efficacy of flavonoids or customizing the diet to enrich for specific flavonoids may be strategies that can be adopted in the future once a better understanding of the interrelationship between flavonoid bioactivity and the gut microbiota is achieved. Finally, the poor bioavailability and solubility of flavonoids makes their use as a potential chemopreventive drug challenging and will require novel strategies to optimize their delivery, dose, and metabolism. As flavonoids have potential anti-tumor effects not limited to colon cancer, advances made in our understanding of the function and metabolism of flavonoids may contribute significantly to the prevention of human cancers in general.

## Figures and Tables

**Table 1:** Select human studies of flavonoids and colorectal cancer.

Type of Food/Flavonoid	Subjects	Type of Study	No. of Subjects	Dose	Length of Study	Outcome	Reference
Anthocyanidin-rich bilberry extract	Ulcerative colitis	Intervention	13	10 mg/mL	6 weeks; 9 weeks follow-up	Reduced NF-κB activation and production of pro-inflammatory mediators in colon biopsies	[137,138]
Anthocyanidin-rich bilberry extract	Colorectal cancer	Intervention	25	0.5–2.0 g	7 days	Reduced cellular proliferation in tumors	[139]
Black raspberry powder	Colorectal cancer	Intervention	20	60 g	1–9 weeks	Decreased methylation of various tumor suppressor gene promoters, increased apoptosis, and decreased surrogate markers of cellular proliferation in colorectal cancer and normal adjacent tissue biopsies	[140]
Apigenin/(-)-epigallocatechin gallate (EGCG)	Polypectomy and resected CRC patients	2-arm intervention	87	20 mg apigenin, 20 mg EGCG	304 years	Decreased recurrence rate of neoplasia (adenoma or CRC)	[141]
Quercetin	Healthy versus CRC patients	Case–control	2664	Quercetin intake estimated based on food frequency questionnaires		Inverse association between proximal, but not distal colon cancer risk and quercetin intake	[186]
Flavonoids/quercetin, myricetin, kaempferol	Healthy	Observational	71,976 women, 35,425 men	Total flavonoid and individual flavonol intake based on food frequency questionnaire		No association between flavonoid intake CRC risk	[185]
Flavonols	History of adenomatous polyp	Randomized intervention trial	1905	Low-fat, high fiber, high vegetable and fruit intake vs. no intervention control questionnaire	4 years	High intake of flavonols was associated with significantly decreased risk of advanced adenoma recurrence	[142]
EGCG	Polypectomy patients	Randomized intervention trial	136	1.5 g green tea extract tablets (52.5 mg EGCG)	1 years	Reduced incidence of adenoma recurrence	[192]
Green tea	Healthy	Intervention		400 mL green tea liquid	2 weeks	Alterations in the gut microbiome, including increased short-chain fatty acid-producing bacteria and decreased pro-inflammatory bacteria	[207]
Flavonoid	Healthy	Observational	477,312	Flavonoid intake based on food frequency questionnaires	Mean follow-up 11 years	No association between total flavonoid intake and risk of CRC or any CRC subtype	[209]

NF-κB: nuclear factor kappa light chain enhancer of activated B cells; CRC: Colorectal cancer.

**Table 2:** Preclinical studies of effect of flavonoids on colon tumorigenesis.

Type of Flavonoid	Class	Dose	Model	Results	Reference
Black raspberry anthocyanin extract	Anthocyanidin	5% or 10% extract, or 3.5 µmol/g and 7.0 µmol/g anthocyanin concentration in diet	Azoxymethane (AOM)/dextran sulfate sodium (DSS) model of C57BL/6 mice	Reduction in colon carcinogenesis; reduction of pathogenic bacteria	[133]
Black raspberry-derived anthocyanins	Anthocyanidin	0.5, 5 and 25 µg/mL	Human colorectal carcinoma cell line HCT116, Caco2, and SW480 cells	Suppressed activity and protein expression of DNMT1 and DNMT3B	[134]
Cyanidin and delphinidin	Anthocyanidin	25–100 µM	Human colorectal carcinoma cell line Caco-2, LoVo/ADR cells	Induced cell death	[128]
Delphinidin	Anthocyanidin	30–240 µM	Human colon cancer HCT116 cells	Induced cell death; inhibition of NFκB	[130]
Dried bilberries or 1 or 10% anthocyanins (ACs)	Anthocyanidin	1 and 10% ACs	Acute and chronic dextrane sodium sulphate (DSS) colitis of Balb/c mice	Amelioration of acute as well as chronic colitis	[131]
Mirtoselect, an anthocyanin mixture from bilberry	Anthocyanidin	0.1% and 0.3% (*w*/*w*) ACs in diet	Apc(Min) mice, a genetic model of human familial adenomatous polyposis	Reduced adenoma load dose-dependently	[135]
Apigenin	Flavone	50–80 µM	Human coloncarcinoma cell lines SW480, HT-29, and Caco-2	Cell-cycle arrest at G2/M; p34(cdc2) and decreased cyclin B1 protein expression	[143]
Apigenin analogs (acacetin, chrysin, kampherol, luteolin, myricetin, naringenin, and quercetin)	Flavone	40–80 µM	Human SW480 and Caco-2 colonic carcinoma cells	G2/M cell-cycle arrest	[144]
Apigenin	Flavone	20–80 µM	HT29-APC cells	G2/M cell-cycle arrest; induction of APC protein expression	[145]
Apigenin	Flavone	10 µM in vitro; 25 and 50 mg/kg in vivo	Human colorectal cancer cells HCT-116, SW480, HT-29 and LoVo; APCMIN+ mice	Reduced polyp numbers; increased p53 activation	[148]
Apigenin and quercetagetin	Flavone	200 µM	Human colorectal colon cancer (SW480) cells	Inhibition of cell proliferation; alter the expression of *bax* and *bcl2* transcription	[149]
Apigenin	Flavone	50 µM	HCT116 human colon cancer cells	Apoptosis- and autophagy-inducing effects	[146]
Apigenin	Flavone	1.5626–100 µM	Colorectal cell lines HT-29 and HCT-15	Pro-oxidative stress induction	[147]
Apigenin	Flavone	25–100 µg/mL	Human gut bacteria	Inhibited *Enterococcus caccae* growth; up-regulated genes involved in DNA repair, stress response, cell wall synthesis, and protein folding	[165]
Quercetin	Flavonol		Human colon cancer COLO320 DM cells	Inhibited cell growth	[170]
Quercetin and rutin	Flavonol	2% quercetin; 4% rutin in diet	Azoxymethane (AOM)-induced colonic neoplasia in mice	Reduced hyperproliferation of colonic epithelial cells and focal areas of dysplasia (FAD) incidence	[175]
Quercetin and rutin	Flavonol	2% quercetin; 4% rutin in diet	AOM-induced colonic neoplasia in mice	Reduced FAD	[178]
Quercetin	Flavonol	25–100 µM	HT-29 and SW480 colon cancer cells	Inhibited cell growth and promoted apoptosis; down-regulated ErbB2/ErbB3 signaling and Akt pathway	[166]
Quercetin	Flavonol	5–50 µM	Caco-2 cells	Induced cell cycle arrest; downregulated expression of cell cycle genes	[168]
Quercetin	Flavonol	100 µM	SW480 cells	Inhibited the transcriptional activity of β-catenin/Tcf	[172]
Quercetin, curcumin, rutin, and silymarin	Flavonol	30,000, 50, 8000, and 5000 p.p.m, respectively	AOM-induced rat colon cancer model	Decreased precancerous lesions and induced apoptosis	[177]
Quercetin	Flavonol	15–40 µM	SW480 cells and clone 26 cells	Inhibition of expression of cyclin D1 and survivin as well as the Wnt/β-catenin signaling pathway	[169]
Quercetin	Flavonol	4.5 g/kg diet	Azoxymethane (AOM)-induced rat colon cancer model	Suppressed formation of early preneoplastic lesions in colon carcinogenesis	[176]
Quercetin	Flavonol	100 µM in vitro; 50–100 mg/kg in vivo	HT-29 colon cancer cells in vitro, and xenografts	Reduced tumor growth and induced apoptosis via 5’-AMP kinase (AMPK) activation and p53-dependent apoptotic cell death	[167]
Quercetin	Flavonol	50 µM	Human colon adenocarcinoma derived cell lines Caco2 and DLD-1	Inhibited cellular proliferation associated with increased expression of the endocannabinoid receptor (CB1-R)	[174]
Quercetin, catechin, and puerarin	Flavonol	0.15 g/L culture	Gut microbiota in vitro cultures	Affected relative abundances of various bacteria	[179]
Quercetin	Flavonol	10 mg/kg	DSS induced colitis model in C57BL/6J mice	Ameliorated colitis	[180]
Epigallocatechin-3-gallate (EGCG)	Catechin	0.01% and 0.1% green tea extract	Azoxymethane-induced colon carcinogenesis in the rat	Inhibited chemical carcinogenesis of the gastrointestinal tract	[206]
Epigallocatechin-3-gallate (EGCG)	Catechin	100 µM	Human colorectal carcinoma HT-29 cells	Inhibited cell growth and induced apoptosis associated with JNK activation	[197]
Epigallocatechin-3-gallate (EGCG)	Catechin	50–300 µM	Human colorectal cancer cell lines HT-29 and HCA-7	Reduced cellular proliferation; inhibition of COX-2 and downregulation of NF-κb, ERK1/2, and Akt pathways	[196]
Epigallocatechin-3-gallate (EGCG)	Catechin	0.1%	AOM-induced rat colon cancer model	Decreased tumor numbers associated with Inhibition of indoleamine 2,3-dioxygenase (IDO) activity	[205]
Epigallocatechin-3-gallate (EGCG)	Catechin	50–150 µM	HCT 116 human colon cancer cells	Reduced histone deacetylase (HDAC) and DNMT protein expression	[203]
Epigallocatechin-3-gallate (EGCG)	Catechin	50–200 µM	5-fluorouracil (5FU)-resistant (5FUR) CRC cells and spheroid-derived CSC (SDCSC) xenograft model	Suppressed Notch1, Bmi1, Suz12, and Ezh2; inhibited tumor growth in a xenograft model	[193]
Epigallocatechin-3-gallate (EGCG)	Catechin	50–150 µM	CIMP+ cell lines	Reduced cell viability; induced cell cycle arrest; Decreased DNMT activity resulting in promoter methylation changes at several cancer-related genes such as retinoid X receptor alpha (RXRα)	[204]
Epigallocatechin-3-gallate (EGCG)	Catechin	10–60 µM	Human colon cancer cell lines DLD-1 and SW480	Inhibition of spheroid formation with suppression of proliferation and Wnt/β-catenin pathway	[194]
Epigallocatechin-3-gallate (EGCG)	Catechin	5–40 µg/mL	Colon adenocarcinoma cells COLO205	Induced chromosome instability and triggered apoptosis and inhibition of cell division	[198]

JNK: c-Jun N-terminal kinase; ERK: extracellular signal-regulated kinase; DNMT: DNA methyltransferase.
